# Effect of the prognostic nutritional index on stent restenosis in patients with chronic coronary syndrome

**DOI:** 10.55730/1300-0144.6082

**Published:** 2025-08-17

**Authors:** Burak KARDEŞLER, Serdal BAŞTUĞ, Kamuran KALKAN, Mehmet ERDOĞAN, Furkan KÜLEKCİ, Muhammed Yunus ÇALAPKULU, Nur BETON, Abdullah Nabi ASLAN, Hafize CORUT GÜZEL, Tahir DURMAZ

**Affiliations:** 1 Department of Cardiology, Ankara Bilkent City Hospital, Ankara, Turkiye; 2 Department of Cardiology, Faculty of Medicine, Ankara Yıldırım Beyazıt University, Ankara, Turkiye; 3 Department of Cardiology, Ankara Mamak State Hospital, Ankara, Turkiye; 4 Department of Cardiology, Burhan Nalbantoğlu Hospital, Lefkoşa, Cyprus; 5 Department of Cardiology, Cleveland Clinic Abu Dhabi, Abu Dhabi, UAE

**Keywords:** Prognostic Nutritional Index, chronic coronary syndrome, in-stent restenosis

## Abstract

**Background/aim:**

In-stent restenosis (ISR) remains a significant clinical challenge in patients undergoing percutaneous coronary interventions. We investigated the association between Prognostic Nutritional Index (PNI) and ISR in patients with chronic coronary syndrome.

**Materials and methods:**

A total of 1007 patients who underwent coronary angiography for a provisional diagnosis of chronic coronary syndrome between March 1, 2019, and July 1, 2022, were included in the study. They were divided into two groups: ISR (n = 395) and non-ISR (n = 612). PNI was calculated using patients’ laboratory values before coronary angiography with the following formula: PNI = (10 × albumin (g/dL)) + (0.005 × total lymphocyte count (per mm^3^)).

**Results:**

The mean age of the patients was 62 ± 10 years, and 75% of the patients (n = 760) were male. The calculated PNI was 42.83 ± 3.53 in the non-ISR group and 44.43 ± 3.74 in the ISR group (p < 0.001). When the validity of the PNI for ISR was statistically examined, the AUC was calculated as 0.64 (0.61–0.68). The cutoff value for PNI was determined to be 44, which predicted stent ISR with a sensitivity of 61% and a specificity of 61%.

**Conclusion:**

Contrary to the findings in the literature, our study revealed a higher Prognostic Nutritional Index (PNI) value in the stent ISR group than in the non-ISR group. The PNI reflects the current nutritional status, whereas stent ISR is an outcome of a chronic clinical condition.

## 1. Introduction

In-stent restenosis (ISR) is described as a reduction in the lumen width following percutaneous coronary intervention (PCI), regardless of whether stent implantation is performed [1]. In patients who do not receive a stent following PCI, ISR typically occurs because of vessel remodeling and elastic recoil. Conversely, in patients with stent implantation, it arises from excessive tissue proliferation in the luminal vessel, known as neointimal proliferation, or the development of a new atherosclerotic process referred to as neoatherosclerosis [1–3].

Previous studies have identified independent risk factors for ISR development as age, sex, diabetes mellitus (DM), smoking, previous bypass surgery, and low left ventricular ejection fraction [4–6]. Inflammation plays a central role in the pathophysiology of ISR. DM accelerates the development of atherosclerosis by causing hyperglycemia and endothelial dysfunction, while the inflammatory process contributes to the acceleration of atherosclerosis through neointimal hyperplasia [7,8].

Nutritional status and composition of dietary components significantly affect inflammation. Evidence suggests that insufficient nutrition increases inflammation, which in turn accelerates atherosclerosis. The early detection and prevention of malnutrition, which accelerates inflammation, can prevent complications associated with atherosclerosis, highlighting the need for reliable parameters to assess nutritional status. The Prognostic Nutritional Index (PNI) is a score that reveals the nutritional-inflammatory status and can be calculated with ease and low cost from serum albumin and lymphocyte values. Contemporary studies have shown that nutritional status assessed by the PNI is an independent prognostic factor in patients with various cardiovascular diseases [9,10].

In this study, we aimed to explore the association between PNI and stent ISR in patients with chronic coronary syndrome (CCS) who had previously undergone PCI.

## 2. Materials and methods

### 2.1. Study design

Our study was designed as a retrospective experimental case-control study conducted at a single center. Ethical approval for our study was obtained on 21 September 2022 (Decision No. E1-22-2920). The study was conducted in accordance with the principles of the Declaration of Helsinki and the Good Clinical Practice Standards. Given the retrospective design of the study, informed consent was waived.

### 2.2. Study population

A total of 1007 patients who underwent coronary angiography (CAG) between March 1, 2019, and July 1, 2022, with a preliminary diagnosis of CCS, were included in the study according to the eligibility criteria. The patients were divided into two groups: those who developed ISR (n = 395) and those who did not (n = 612). The study involved individuals aged 18 years and older who had previously undergone CAG for any reason and had a coronary artery stent in place, as well as those who underwent elective coronary intervention for CCS.

The exclusion criteria were as follows: having acute coronary syndrome and having undergone CAG; symptomatic heart failure with a left ventricular ejection fraction (EF) < 40%; congenital cardiac disease; severe valvular heart disease; severe liver failure; chronic renal insufficiency (GFR < 30 mL/min/1.73 m^2^); body mass index > 35 kg/m^2^; a history of familial dyslipidemia or familial hypertriglyceridemia; a history of malignancy; autoimmune diseases; connective tissue disorders; and active infections.

ISR was described angiographically as the presence of >50% stenosis observed inside the stent or at the proximal and distal edges up to 5 mm from the stent borders. This condition was considered an in-stent ISR [11].

The medical records of the study participants were retrospectively reviewed using the national health registry system and pharmacy records, and demographic data were collected. Two interventional cardiologists independently evaluated the patients’ previous CAG and PCI. The degree of restenosis was visually estimated following standard clinical practice, and the decision for CAG in patients with CCS was made in accordance with the current guidelines. All data, including lesion characteristics, stent types used, and ISR, were recorded.

Complete blood count evaluations were performed using the XN-1000 device (Sysmex, Kobe, Japan), whereas other biochemical parameters were analyzed using the COBAS C-501 device (Roche, Mannheim, Germany) in the biochemistry laboratory. The PNI was calculated using the formula: (10 × albumin (g/dL)) + (0.005 × total lymphocyte count (per mm^3^)) based on the patients’ laboratory values obtained prior to coronary angiography.

### 2.3. Statistical analyses

The analyses were performed using IBM SPSS Statistics (version 26.0; IBM Corp., Armonk, New York, USA) and Microsoft Excel for Mac. The spread of variables was determined using the Kolmogorov–Smirnov test and histogram curves. For groups with normally distributed numerical variables, Student’s t-test was applied, while the Mann–Whitney U test was applied to compare numerical variables that were not normally distributed. Numerical variable results are presented as the mean ± standard deviation and median (25th–75th percentiles). Categorical variables were analyzed using the chi-square test or Fisher’s exact test, as appropriate, and are presented as counts (percentages). Correlation analysis between the degree of stenosis and the medical characteristics of patients with ISR was performed using Spearman’s correlation analysis, with the correlation coefficient (r) presented. Receiver Operating Characteristic (ROC) curve analysis was performed to predict stent ISR and determine cutoff values for PNI levels. Sensitivity, specificity, and area under the ROC curve (AUC) are presented with 95% confidence intervals (CI). Based on the cutoff values obtained from the ROC curve, the patients were divided into high and low PNI groups. The cutoff value for the PNI was calculated to be 44. Cumulative risk ratios were computed using the Kaplan-Meier method, and the low and high PNI groups were compared using the log-rank (Mantel-Cox) test. Subsequently, time-dependent Cox proportional hazards regression analysis was conducted for stent ISR. The clinical, demographic, laboratory, and procedural parameters thought to be associated with ISR, as well as the PNI value, were applied to the univariate Cox regression model. Unadjusted subgroup data obtained from this analysis are presented using a forest plot. Variables with a p-value < 0.10 in the univariate Cox regression were included in the multivariate model to identify independent predictors of ISR. The proportional hazard assumption was evaluated using Schoenfeld residuals, and no significant violations were detected. The findings from the regression analysis are presented with 95% confidence intervals (CI) and hazard ratios (HR). A p-value of < 0.05 was contemplated statistically significant.

## 3. Results

Seventy-five percent of the study patients (n = 760) were male, with a mean age of 62 ± 10 years. In patients with ISR, the proportions of males and smokers were significantly higher than those in the non-ISR group (p = 0.031 and p < 0.001, respectively). When comparing medication use between the two groups, the use of beta-blockers and statins was significantly lower in the ISR group than in the non-ISR group (p = 0.011 and p = 0.009, respectively). The baseline demographic, clinical, and laboratory characteristics and medication usage information for the groups with and without ISR are presented in Table 1.

In the non-ISR group, the PNI value was 42.83 ± 3.53, whereas the PNI value in the ISR group was 44.43 ± 3.74, with a statistically significant difference among the groups (p < 0.001). Significant differences were observed between the groups in terms of total protein, creatinine, triglyceride, and LDL cholesterol levels (p < 0.05). Among the complete blood count parameters, hematocrit, white blood cell count, and neutrophil count were significantly different between the two groups (Table 1).

The type of stent insertion was significantly different between the two groups (p = 0.010). As the length and diameter of the initially implanted stent increased, the ISR significantly increased (p < 0.001, p = 0.031). The median time since first stent implantation was 40 months (range: 24–68 months) in the non-ISR group and 61 months (range: 35–88 months) in the ISR group, with a statistically significant difference between the groups (p < 0.001).

Regarding the treatment administered to patients who developed ISR, balloon angioplasty within the stent was the most commonly applied method (60%), followed by medical follow-up in 22%, balloon and stent implantation in 21%, and coronary artery bypass graft surgery in only 12% of patients (Table 2).

**Figure 1:**
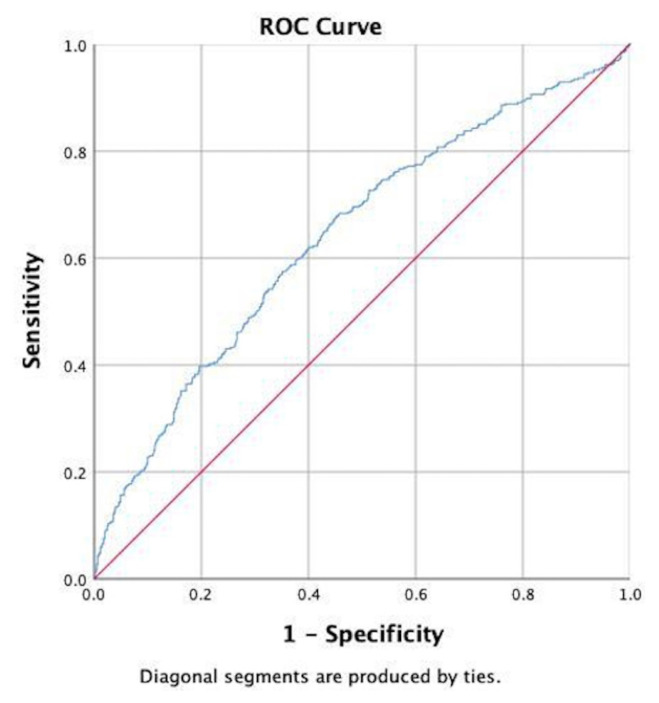
ROC curve analysis demonstrating the predictive accuracy of prognostic nutritional index levels for the prediction of in-stent restenosis (AUC = 0.64, 95% CI: 0.61–0.68, p < 0.001).

**Figure 2:**
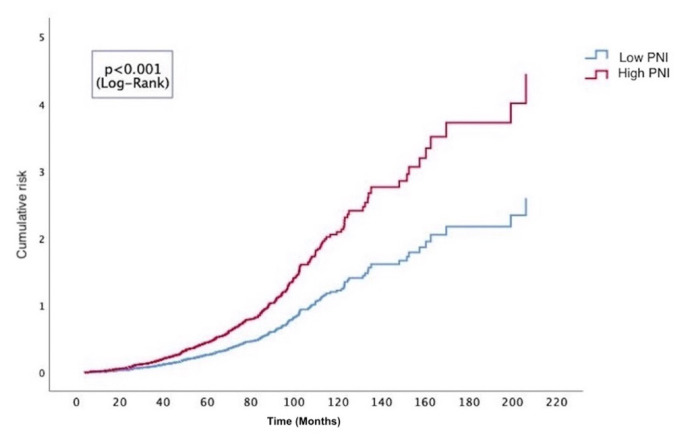
Kaplan-Meier curves of cumulative risk of low and high PNI values according to the cut-off value. (Abbrevations: PNI; prognostic nutritional index)

The PNI value in the ISR group was evaluated as a predictor of ISR using the ROC curve analysis. The cutoff value for PNI was 44, which predicted stent ISR with 61% sensitivity and 61% specificity (AUC 0.64, 0.61–0.68; p < 0.001) (Figure 1). Based on the ROC analysis, the cumulative risk curve estimated by the Kaplan–Meier method for the low and high PNI groups based on the cutoff value of 44, revealed that the group with high PNI (>44) had a higher risk throughout the median 86-month follow-up period (95% CI: 80–91; (p < 0.001, log-rank) (Figure 2).

**Figure 3:**
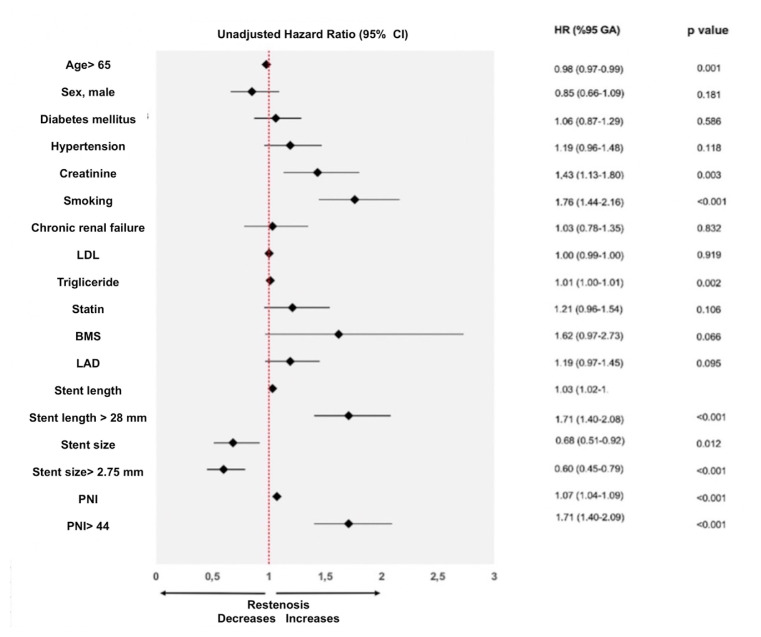
Forest plot graph shows hazard rates for stent restenosis in defined subgroups. (Abbrevations: LDL; low density lipoprotein, BMS; bare metal stent, LAD; left anterior descending artery, PNI; prognostic nutritional index)

In the time-dependent multivariate Cox proportional hazard regression model, the independent prognosticators of ISR were identified as smoking (HR: 1.61, 95% CI: 1.30–1.98, p < 0.001), creatinine level (HR: 1.48, 95% CI: 1.14–1.91, p = 0.003), type of stent used (HR: 2.54, 95% CI: 1.40–4.62, p = 0.002), length of stent (HR: 1.02, 95% CI: 1.01–1.04, p < 0.001), diameter of stent (HR: 0.59, 95% CI: 0.42–0.84, p = 0.003), and PNI value (HR: 1.05, 95% CI: 1.02–1.08, p = 0.001) (Table 3). The subgroup data obtained from this analysis is shown as a forest plot (Figure 3).

## 4. Discussion

The key finding of our study was that as the PNI value increased, the incidence of ISR also increased. Additionally, increased stent length, reduced stent diameter, consumption of bare-metal stents (BMS), elevated creatinine levels, and smoking were identified as independent predictors of ISR. This study aimed to investigate the association between ISR and PNI, an indicator of current nutritional status, in patients with chronic coronary syndrome (CCS) who had previously undergone PCI for acute or CCS diagnoses. To our knowledge, this study represents the largest cohort to date evaluating the relationship between PNI and ISR. The strength of our study lies in the larger number of patients who received drug-eluting stents (DES), setting it apart from previous research.

To more effectively utilize emerging therapies to prevent ISR, it is crucial to identify the underlying risk factors and take preventive measures accordingly. Risk factors for ISR have been previously defined, with the presence of diabetes mellitus (DM) and acute inflammation identified as the most significant clinical risk factors [12]. Hyperglycemia associated with DM leads to endothelial dysfunction and accelerates the development of atherosclerosis, whereas inflammatory processes contribute to atherosclerosis development through neointimal hyperplasia [8,13].

Nutritional status and composition of dietary components significantly influence inflammation. Inflammation is believed to increase in cases of improper or insufficient nutrition, thereby accelerating atherosclerosis. Parameters that reliably reflect nutritional status can be used to predict inflammatory activity and help prevent potential acute complications. For this purpose, the PNI, a real-time indicator of nutritional status, was employed.

However, it should be noted that while the PNI value reflects the current status, inflammation-driven acceleration of atherosclerosis is a chronic process [14]. Inflammation may link inadequate nutrition to cardiovascular outcomes, and previous studies have shown that atherosclerosis, intimal hyperplasia, and inflammation contribute to ISR pathophysiology [15].

Unlike other studies, our study found an inverse relationship between PNI and ISR in patients with CCS. We believe this could be attributed to the fact that the PNI value reflects the current nutritional status. Nutritional status has been assessed in prior studies using various nutritional scores, such as the Controlling Nutritional Status (CONUT) score and the Mini-Nutritional Assessment (MNA) score. In our study, we evaluated nutritional status using the PNI value. The PNI was originally described by Buzby et al. in 1980 as an unbiassed nutritional screening tool [16]. The PNI value reflects the recent nutritional status and can be easily estimated from serum albumin and total lymphocyte counts. This method is widely accessible and inexpensive. The lymphocyte and albumin levels used in the PNI calculation can be influenced by various factors, including infection, malignancy, and medication use. The PNI has previously been used as a prognostic indicator in heart failure, malignancies, and STEMI and NSTEMI patients [9,10,17]. Although the cross-sectional and current values of PNI can provide insights into acute clinical pathologies, as shown in our study, no significant correlation was established between PNI values and clinical events in chronic pathologies.

Based on previous studies that used PNI, Balun et al. found a negative correlation between PNI and ISR, indicating that as PNI decreases, the risk of ISR increases, which is statistically significant [18]. In that study, the sample size was smaller, and most of the stents used were bare-metal stents. In contrast, in our study, the majority of stents used were drug-eluting stents (DES); as a result, the rate of stent ISR was lower. Nakagomi et al. demonstrated that malnutrition in patients with chronic heart failure is linked to carotid atherosclerosis [19]. Chen et al. demonstrated the prognostic significance of PNI in patients with STEMI [9]. However, in our study, contrary to the literature, a positive correlation was observed between the PNI and ISR. This clinical finding might be attributed to the chronic physiology of ISR and atherosclerosis, whereas PNI reflects acute nutritional status. This observation may be explained by the shorter follow-up in the non-ISR group and the generally better clinical condition of the CCS patients compared with those with acute coronary syndrome, malignancy, or heart failure. Additionally, in acute coronary events, corticosteroid levels increase, leading to lymphocytopenia, and a higher lymphocyte count is associated with an appropriate immune response and stable inflammatory condition. Lymphocytopenia is self-reliantly associated with major adverse cardiac events and mortality following myocardial infarction [20]. However, in our study, the lymphocyte levels showed no significant differences among the groups.

The PNI value provides real-time information about the disease, which is valuable in acute clinical pathologies. However, in chronic diseases, particularly in conditions associated with accelerated inflammation, there is a need for other markers that can provide more insight. A similar clinical scenario has been observed in patients with diabetes mellitus. Although elevated glucose levels are important in acute pathologies, HbA1c, which reflects long-term glycemic control, has been used as an indicator of mortality and prognosis in numerous studies. Zhao et al. found an association between HbA1c levels and chronic coronary syndrome (CCS) [21].

The unexpected observation of a higher PNI in the ISR group, contrary to previous studies linking a lower PNI to adverse cardiovascular outcomes, warrants deeper consideration. One possible explanation is that certain components of the PNI, such as serum albumin or lymphocyte count, may have been influenced by unmeasured confounders including corticosteroid use, occult inflammatory or autoimmune conditions, or variations in hydration status. These factors may artificially elevate the PNI despite the presence of subclinical vascular inflammation.

Moreover, differences in baseline characteristics between the ISR and non-ISR groups, such as glycemic control, statin use, renal function, and adherence to dietary recommendations, could have influenced both nutritional status and ISR development. The cross-sectional nature of the study precludes the assessment of the causality or directionality of these associations.

Comparisons with previous studies reporting an inverse PNI–cardiovascular risk relationship also highlight key methodological differences. To illustrate, earlier studies often focused on acute coronary syndromes, heart failure, or postoperative outcomes, where a low PNI reflected catabolic stress and poor prognosis. ISR is a chronic vascular healing disorder that may involve distinct pathophysiological mechanisms. Differences in stent type (e.g., first- vs. second-generation drug-eluting stents), patient selection (elective vs. urgent cases), and follow-up duration may also contribute to divergent findings.

These observations raise an important conceptual question: is PNI, as a static index, an appropriate biomarker for a long-term process such as ISR? While PNI is convenient and widely available, it may not fully capture the chronic low-grade inflammatory state driving neointimal proliferation. Alternative indices such as the CONUT score or direct measurement of inflammatory cytokines (e.g., IL-6 and TNF-α) may offer more specificity in capturing the pathobiology of ISR. Future prospective studies incorporating such markers along with serial PNI assessments could help clarify these complex relationships.

Although we identified a statistically significant association between PNI and ISR, the discriminative ability of PNI was modest, as reflected by the ROC curve analysis (AUC = 0.64), with sensitivity and specificity values of 61%. These findings indicate that while the PNI may provide some prognostic insight, it is unlikely to be sufficient as a standalone biomarker for ISR prediction or clinical decision-making. In clinical practice, risk stratification tools must offer a higher discriminatory power to justify their routine use.

Therefore, the current predictive performance limits the clinical applicability of PNI in isolation. To enhance risk prediction, PNI can be integrated into a multivariable model incorporating procedural characteristics (e.g., stent type, residual stenosis, lesion length), metabolic parameters (e.g., HbA1c, LDL-C), and established inflammatory markers (e.g., hs-CRP, neutrophil-to-lymphocyte ratio). This composite approach may improve the identification of patients at a higher risk of ISR and guide more personalized surveillance or secondary prevention strategies. Further studies are required to develop and validate integrative predictive models.

Our study had several limitations. First, the PNI value we used reflects the current nutritional status. However, serial measurements of PNI components and longitudinal follow-up of PNI values have not been performed. The inability to show dynamic changes in PNI values and chronic nutritional status limits the utility of PNI in predicting ISR. Future studies should consider serial PNI assessments to better characterize the temporal dynamics of inflammation and nutrition. Moreover, combining PNI with established chronic inflammatory markers, such as high-sensitivity C-reactive protein (hs-CRP) and interleukin-6, or glycemic indices, such as HbA1c, could enhance risk stratification for ISR.

Second, our study followed a single-center retrospective cohort design, highlighting the need for a multicenter cohort design in future research. Third, there were differences between the groups in terms of the time elapsed since the initial stent implantation and the interventional cardiologist who performed the procedure. Finally, we were unable to assess the continuity of medical treatments or changes in risk factors from the first stent implantation to angiography. While we collected prescription data from the medical records, we acknowledge that actual medication adherence, appropriate dosing, and dietary compliance are important confounding factors that could influence our results. Fully controlling these variables is challenging in a retrospective study design. In addition, data on minimum lumen area and residual stenosis after the index procedure were unavailable due to the retrospective design. Despite multivariate adjustment, residual confounding may persist, especially regarding medication adherence and stent characteristics (e.g., polymer type and drug-coating durability), which could not be captured retrospectively. Prospective studies with standardized procedural assessments are needed to further clarify the relationship between nutritional indices, such as PNI and ISR, while controlling for such procedural variables.

## 5. Conclusion

We found that the PNI, which reflects the current nutritional status, is insufficient for use in chronic clinical conditions, such as ISR. In diseases associated with chronic inflammation, such as ISR and atherosclerosis, there is a need for new biomarkers that can reflect the patients’ chronic nutritional status. To clarify the association between ISR and PNI values in patients with CCS, long-term prospective studies are needed along with new research that can measure dynamic changes in PNI values.

## Figures and Tables

**Table 1:** Comparison of baseline demographic and comorbid conditions between groups with and without restenosis.

Variables	No in-stent restenosis (n:612)	In-stent restenosis (n:395)	p-value
Age, years	62±10	63±10	0.497
Sex, male, n (%)	447 (73)	313 (79)	0.031
Diabetes, n (%)	241 (39)	170 (43)	0.249
Hypertension, n (%)	398 (65)	282 (71)	0.035
Hyperlipidemia, n (%)	177 (29)	133 (34)	0.111
Smoking, n (%)	145 (24)	159 (40)	<0.001
Chronic renal failure, n (%)	83 (14)	61 (15)	0.405
Statins, n (%)	509 (83)	302 (76)	0.009
Glucose (mg/dL)	99 (88–120)	109 (92–142)	<0.001
Albumin (mg/dL)	42.8±3.5	44.4±3.7	<0.001
Total protein (mg/dL)	66 (63–69)	72 (69–74)	<0.001
Urea (mg/dL)	34 (28–42)	35 (29–43)	0.379
Creatinine (mg/dL)	0.93±0.26	1.00±0.43	0.004
LDL cholesterol (mg/dL)	82 (65–108)	95 (73–117)	<0.001
HDL cholesterol (mg/dL)	40±10	40±12	0.541
Triglyceride (mg/dL)	145 (102–200)	156 (112–232)	0.001
Hemoglobin (g/dL)	13.9±5.4	14.0±1.6	0.633
White blood cell (K/uL) × 10^3^	7.56 (6.36–8.89)	7.90 (6.69–9.48)	0.004
Neutrophil (K/uL) × 10^3^	4.58 (3.75–5.56)	4.82 (3.90–6.14)	0.002
Lymphocyte (K/uL) × 10^3^	2.00 (1.54–2.52)	2.00 (1.54–2.50)	0.898
Platelet (K/uL) × 10^3^	251±69	260±78	0.054
Prognostic nutritional index	42.83±3.53	44.43±3.74	<0.001

Abbreviations: LDL; low-density lipoprotein, HDL; high-density lipoprotein.

**Table 2:** Stent characteristics in groups with and without restenosis.

Variables	No in-stent restenosis (n:612)	In-stent restenosis (n:395)	p-value

Stented coronary artery, n (%)			
LAD	335 (55)	172 (44)	0.001
CX	109 (18)	75 (19)	
RCA	168 (27)	148 (37)	

Stent type, n (%)			
Bare-metal stent (BMS)	8 (1)	15 (4)	0.010
Drug-eluting stent (DES)	604 (99)	380 (96)	

Stent length (mm)	24±7	28±8	<0.001

Stent diameter (mm) (Median-IQR)	3.00 (2.75–3.00)	3.00 (2.75–3.00)	0.017

Stent diameter (mm) (Mean±SD)	3.28±2.75	2.97±0.38	0.031

Time since implantation (months)	40 (24–68)	61 (35–88)	<0.001

Abbreviations: LAD, left anterior descending artery; CX, circumflex artery; RCA, right coronary artery; BMS, bare metal stent; DES, drug-eluting stent.

**Table 3:** Univariate and multivariate cox regression analysis for restenosis.

Variables	Univariate		Multivariate		
	Hazard ratio	95% confidence interval	p-value	Hazard ratio	95% confidence interval
Age	0.98	0.97–0.99	0.001	0.99	0.98–1.01
Sex	0.85	0.66–1.09	0.181	-	-
Diabetes	1.06	0.87–1.29	0.586	-	-
Hypertension	1.19	0.96–1.48	0.118	-	-
Smoking	1.76	1.44–2.16	<0.001	1.61	1.30–1.98
Chronic renal failure	1.03	0.78–1.35	0.832	-	-
LDL cholesterol	1.00	0.99–1.00	0.919	-	-
Triglyceride	1.01	1.00–1.01	0.002	1.00	1.00–1.01
Statins	1.21	0.96–1.54	0.106	-	-
Creatinine	1.43	1.13–1.80	0.003	1.48	1.14–1.91
Stent type (BMS vs DES)	1.62	0.97–2.73	0.066	2.54	1.40–4.62
Stented artery (LAD vs non-LAD)	1.19	0.97–1.45	0.095	1.12	0.91–1.38
Stent length	1.03	1.02–1.04	<0.001	1.02	1.01–1.04
Stent diameter	0.68	0.51–0.92	0.012	0.59	0.42–0.84
PNI	1.07	1.04–1.09	<0.001	1.05	1.02–1.08

Abbreviations: LDL, low-density lipoprotein; BMS, bare metal stent; DES, drug-eluting stent; LAD, left anterior descending artery; PNI, prognostic nutritional index.
